# Primary and Secondary Amputation—Which?

**Published:** 1880-12

**Authors:** 


### PRIMARY AND SECONDARY AMPUTATION—WHICH?

THIS question is variously answered by different surgeons. Prof. Richet (*Union Med.—Medical Times and Gazette*) tells us that in the earlier part of his career he practiced primary amputations. As a result most of his patients died. On communicating his ill fortune to Malgaigne, the latter told him that he had gone through the same experience. He said that investigation had convinced him that the mortality from primary amputations was eighty-six per cent. The reasons for this mortality Prof. Richet gives as follows: "Whenever an individual has undergone a violent injury, his nervous system is greatly shaken by it, his pulse is depressed and his temperature notably lowered. Further, the circulation is delayed to such a point that the soft parts soon assume violaceous color and then become gangrenous, at least in places. Soon there supervene intramuscular tumefactions and subcutaneous emphysema, the percursor of sphacelus. In these cases we have functional disturbance of the nervous system and of the circulatory system, and to these primary amputation adds the shock of mutilation, and a considerable moral disturbance. If, on the other hand, it is decided to make a secondary operation, there will necessarily be a considerable number of fatal cases; but these belong to the class that would have died under an immediate amputation. Others, fewest in number, will traverse the first accident with success. The nervous system recovers itself little by little; the circulation regains strength, and the temperature rises; and, as a consequence, two or three days afterwards, normal inflammatory phenomena begin to appear. Is this then the moment at which amputation will have greatest chance of success? Not yet. Such at least was the opinion Velpeau and of Roux, with which Richet coincides. They never operated before the fifth or sixth day, and this is also his habit of proceeding."—*Detroit Lancet.*

